# Therapeutic Notes

**Published:** 1888-09

**Authors:** H. C. Morrow


H. C. MORROW, M. D.

---

### THERAPEUTIC NOTES.

H. C. MORROW, M. D.

Visions on closing the eyes: Apis, *Arg-nit*,. Arsen., Atrop., BELL., *Bry.*, CALC-C., Camph., *Caust.*, Chloral., *Cinch.*, Cocc., Euphra., Gels. (clinical), Graph., Hell., Igna., *Lach.*, Led., Lycop., Nat-m., Nat-ars., Petiv., Plumb., Puls., *Samb.*, Scroph., Sep., *Spong.*, Stram., Sulph., Tarent., Thu.

Are there any others? The authority for each of the above can be given.

Stool smelling like rotten eggs: Asclep. tub., Arsen. alb., *Calc.*, Carls., *Cham.*, Fagop., Hep-s., *Psor.*, *Sulph.*, Sulph-ac., Wies. Any more?

